# Prevalence of tuberculosis among People Who Use Drugs 2000–2024: a systematic review and meta-analysis

**DOI:** 10.3389/fpubh.2025.1635053

**Published:** 2025-10-03

**Authors:** W. S. Ngowi, O. O. Mandizadza, Minyan Wang, W. T. Shao, Conghua Ji

**Affiliations:** School of Public Health, Zhejiang Chinese Medical University, Hangzhou, China

**Keywords:** tuberculosis (TB), People Who Use Drugs (PWUD), people who inject drugs (PWID), meta-analysis, system evaluation

## Abstract

**Background:**

People who use drugs (PWUD) are at a higher risk of developing tuberculosis (TB); however, there is no clear evidence to determine the prevalence of TB in this group accurately. This study aimed to systematically review the prevalence of tuberculosis (TB) among PWUD across studies conducted in different countries.

**Methods:**

An electronic search for original articles on the prevalence of TB among People Who Use Drugs (PWUD) was conducted in PubMed, Web of Science, and Science Direct. The reference lists of included studies were manually screened to identify omitted eligible studies. Extracted data were imported into a Microsoft Excel sheet. The random-effects model was employed to estimate the pooled prevalence of TB among People Who Use Drugs (PWUD), with effect size (ES) reported as point estimates and 95% confidence interval (CI). Subgroup analysis and sensitivity tests were also performed. All analyses were performed in STATA version 18.

**Results:**

A total of 2,085 articles were retrieved from the search. After removing duplicates, screening titles and abstracts, and excluding non-eligible articles, 23 studies involving 164,121 patients met the inclusion criteria for the analysis. The average estimated prevalence of tuberculosis among People Who Use Drugs (PWUD) was 25% (95% CI: 0.21, 0.35). The prevalence of tuberculosis among PWUD was higher in Asia (32%) and North America (28%) compared to South America (10%) and Africa (5%). Additionally, the average prevalence of tuberculosis among PWUD was 36% in case-control studies, 26% in cohort studies, and 23% in cross-sectional studies.

**Conclusion:**

This review highlighted a high pooled prevalence of tuberculosis among People Who Use Drugs (PWUD), which varies across continents, study types, drug categories, and assessment tools. This emphasizes the need to integrate screening and prevention strategies into programs for PWUDs, address systemic inequities to reduce transmission, improve data reporting, and conduct more research in under-reported regions such as Africa and South Asia.

**Systematic review registration:**

identifier CRD42024564181.

## Introduction

Over the years, tuberculosis (TB) has remained a significant public health concern. With the implementation of preventive measures and effective treatments, the incidence of TB and related deaths has significantly decreased over time. However, TB continues to be one of the leading causes of death worldwide (1). In 2023, the World Health Organization (WHO) estimated 10.8 million deaths from TB, including 6.3 million men, 3.6 million women, and 1.3 million children. More than 1.25 million individuals were projected to die from TB, including 161,000 co-infected (1). In the previous years, many TB cases have been linked to factors such as undernourishment, HIV infection, diabetes, alcohol consumption, cigarette smoking, and other substance use (1).

Drug use refers to excessive or addictive consumption of drugs for non-medical reasons, despite potential social, psychological, and physical problems that may result. The most prevalent category, with a long history of abuse, includes opioids such as heroin, hallucinogens, barbiturates, cocaine, various forms of cannabis, and alcohol. It is widely recognized that HIV/AIDS is strongly associated with the prevalence of tuberculosis. In 2023, approximately 161,000 people died from HIV-associated TB. The proportion of notified TB patients with a documented HIV test result in 2023 was 80%, maintaining the same level as in 2022, but up from 76% in 2021. The WHO African Region bears the highest burden of HIV-associated TB. Overall, in 2023, only 56% of TB patients known to be living with HIV were on antiretroviral therapy (ART) (2). People Who Use Drugs (PWUD) face a high risk of contracting or spreading viral infections such as human immunodeficiency virus (HIV), viral hepatitis B (HBV), viral hepatitis C (HCV), and other sexually transmitted infections (STIs). This risk primarily arises from participation in high-risk behaviors such as sharing needles, engaging in risky sexual practices, including exchanging sex for money or drugs, having multiple sexual partners, and inconsistent condom use. A study showed that 9 of the 52 patients (17.3%) had RR-TB. Of the 52 TB+ subjects, 8 (15.4%) also had HIV. Of the 522 people having an HIV test result, 29 tested positive for HIV, indicating a prevalence of 5.6% (95% CI [3.8%−8.0%] (3). Additionally, Tuberculosis (TB) is a significant public health challenge, especially among PWUD, who are more vulnerable to the disease (4). The connection between drug use and TB is linked to late detection of disease progression, poor adherence to treatment, drug resistance, and greater community transmission (5). A recent study in Ukraine found that 228 People Who Inject Drugs (PWID) were infected with TB (6). Moreover, in several U.S. regions, the prevalence of TB among PWID ranges from 15-39% (7). A study in Ivory Coast also reported a prevalence rate of 7.7% in this population (3). Research indicates that TB prevention, diagnosis, and treatment for PWUD have been neglected in global health efforts and require urgent attention (6). Drug use among people with TB significantly contributes to poor treatment outcomes or failure (8). Compared to TB patients who do not use drugs, People Who Use Drugs (PWUD) are more prone to treatment failure, relapse, and death. Despite these impacts, there is currently no comprehensive evidence on the average prevalence of TB among PWUD globally.

This study aims to synthesize existing evidence and estimate the prevalence of TB among PWUD. Additionally, we seek to identify factors associated with TB infection among PWUD. The findings will highlight the current burden of TB in this population group, and guide public health practitioners in policymaking.

## Method

The study was registered in the Prospective Register of Systematic Reviews (PROSPERO): CRD42024564181.

### Search strategy

Two independent authors systematically searched PubMed, Web of Science, and Science Direct for articles reporting tuberculosis among People Who Use Drugs (PWUD) from the database inception until January 21, 2025. We combined Medical Subject Headings (MeSH) terms and free-text keywords, including “tuberculosis, pulmonary,” “drug users,” and “drug use”. Furthermore, the references of included studies were also manually checked to ensure no relevant articles were missed during the search.

### Study selection

The retrieved literature was imported into a reference manager (EndNote 20.2.1 Clarivate Analytics, Philadelphia, USA) for duplicate removal and screening. Two independent researchers reviewed study titles and abstracts, followed by a full-text review of potentially eligible studies based on the inclusion and exclusion criteria. Any discrepancies were resolved through discussion, with a third researcher providing adjudication if needed.

### Inclusion criteria

Studies were included if the study population involved People Who Use Drugs (PWUD) with Tuberculosis. Studies that reported TB prevalence based on screening tests (e.g., TST results or findings on CXR), diagnostic measures (e.g., positive sputum smear or culture, or clinical diagnosis) for latent or active TB. Additionally, only original studies with primary evidence were considered.

### Exclusion criteria

Studies were excluded if they lacked data on People Who Use Drugs (PWUD) with TB. Additionally, secondary studies (reviews and meta-analyses) and those not published in English were excluded.

### Screening

First, we excluded studies conducted before 2000 due to advancements in TB diagnostics and shifts in substance use patterns. Furthermore, we also removed duplicates. We screened titles and abstracts to eliminate irrelevant studies. Then, we assessed the full texts of studies that met the inclusion criteria. All steps were performed independently by two investigators.

### Data extraction

We extracted relevant data from each included study and organized it in a Microsoft Excel spreadsheet. The following study characteristics were collected: first author, title, publication date, study type, country, total patients (People Who Use Drugs, PWUD), PWUD who have TB, crack cocaine use, heroin use, opioid use, and injecting drugs (PWID).

### Quality assessment

All included studies were observational (cross-sectional, cohort, and case-control). The quality of the articles was assessed using the modified Newcastle Ottawa Scale (NOS) for cohort studies and a modified version for cross-sectional studies (9–11).

## Statistical analysis

We employed a random effects model to account for expected heterogeneity between studies, which varied in designs, types of drugs used, population groups and geographical locations. Therefore, it provides a more conservative and generalized estimate of the pooled prevalence in our study (12).

The heterogeneity of the study was evaluated using the *I*^2^ statistic (*I*-squared variation in ES attributable to heterogeneity), which describes the percentage of variation between studies (13). An *I*^2^ value of 0% indicates no observed heterogeneity, 25–50% is considered low to moderate heterogeneity, and above 75% signifies high heterogeneity (14). Data analysis was performed in STATA version 18.0 using the “meta prop” command. When significant heterogeneity was present, subgroup analyses were conducted based on study type, WHO region, and type of abused drugs to identify potential sources of heterogeneity.

## Results

### Study selection

**Figure 1:**
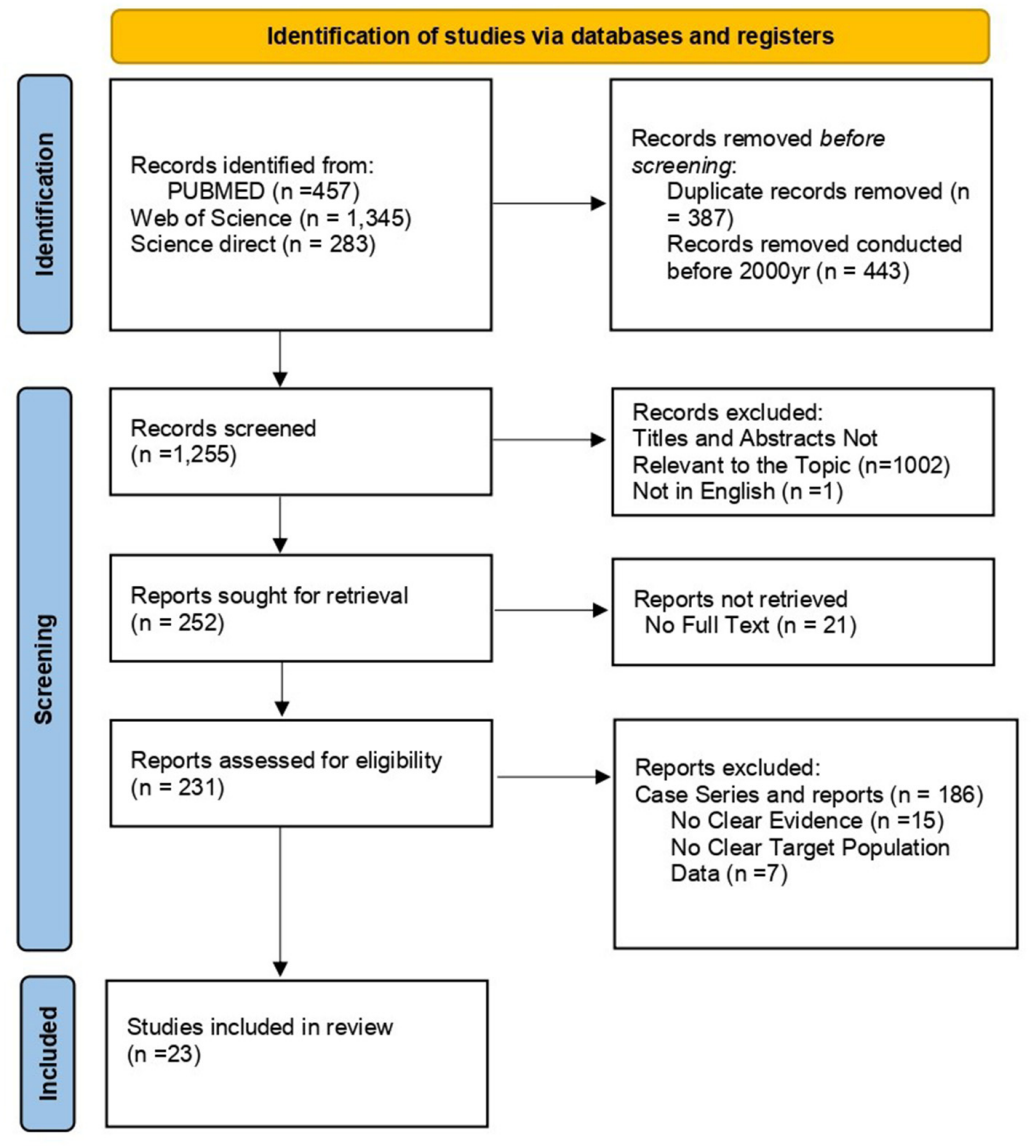
Prisma flow chart.

A total of 2,085 articles were identified through an initial database search. A total of 387 duplicates were removed. After screening titles and abstracts, 45 studies were found eligible, and the full texts of the articles were assessed. Among these, only 23 articles met the inclusion criteria. More details are shown in the PRISMA flow chart presented in Figure 1.

### Study characteristics

A total of 23 studies (13 cross-sectional studies, 7 cohort studies, 2 randomized controlled trials, and 1 case-control study) were included. Among these, 16 studies reported results on PWID (4, 15–24), 9 studies involved opioid users (4, 17, 19, 21, 22, 25–27), 4 studies focused on heroin users (4, 25, 28, 29), and 8 studies included crack cocaine users (4, 17, 19, 21–23, 25, 30, 31). The total number of participants across all studies was 164,121. The studies were conducted in Europe (6 studies), Africa (2), Southeast Asia (3), North America (4), and South America (2). The included studies were published between 2000 (18) and 2021 (4). Various studies used different indicators to report the socio-demographic characteristics of the participants.

### The pooled prevalence rates

**Figure 2:**
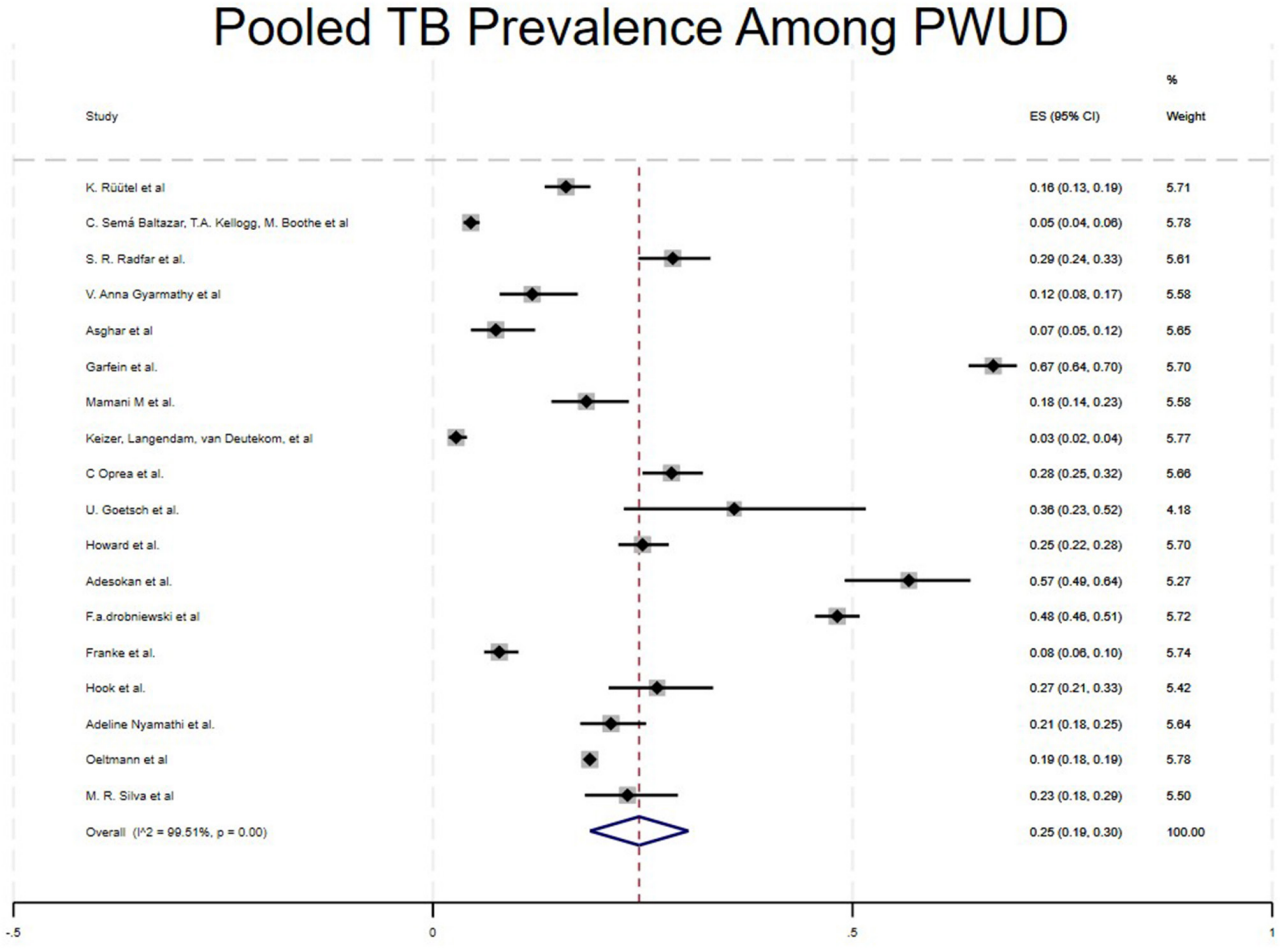
Overall pooled TB prevalence among PWUD.

The pooled prevalence of tuberculosis among People Who Use Drugs (PWUD) across 23 studies was 25% (95% CI: 19%−30 %). The studies with greater weight contributed most to the pooled estimate (16, 22, 26) shown in Figure 2.

### Subgroup analysis

Subgroup analyses were conducted to identify potential sources of heterogeneity and to detect significant trends and factors associated with TB among People Who Use Drugs (PWUD). We examined subgroups based on study type, WHO regions, and drug types.

- *Type of Study*.

**Figure 3:**
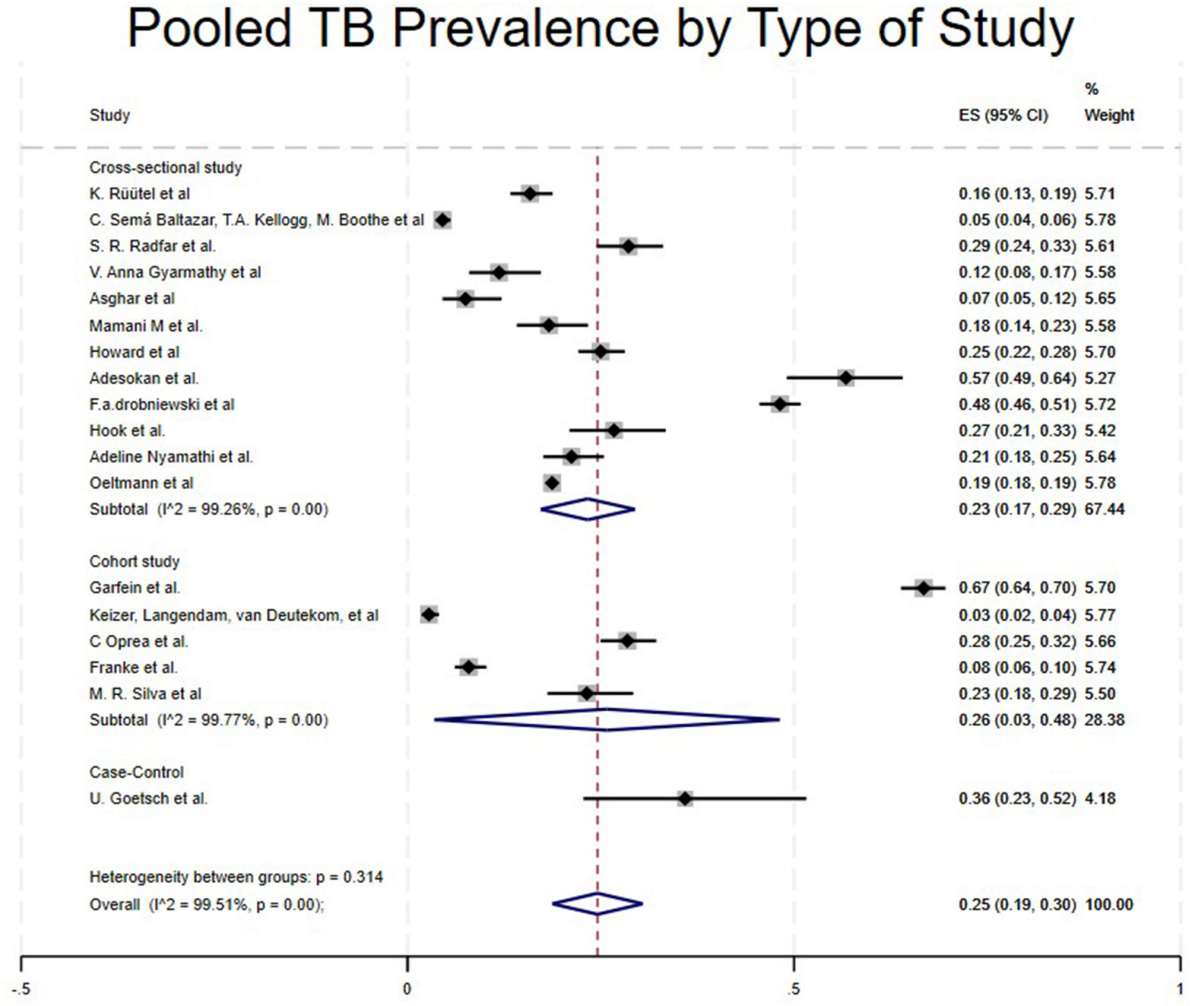
Pooled TB prevalence by type of study.

Case control studies reported a notably high prevalence of 36% (95% CI: 23%−52%) (5). Cohort studies reported a pooled prevalence of 26% (95% CI: 3%−48%) (16, 18, 29, 31, 32), while cross-sectional studies reported a pooled prevalence of 23% (95% CI: 17%−29%) (4, 15, 17, 21–28, 30) as shown in Table 1 and Figure 3. Randomized controlled trials and a few studies with a prevalence equivalent to 1 were excluded from the forest plot results (19, 20, 33, 34).

**Table 1:** Prevalence based on different subgroups.

**Subgroups**		**Number of studies**	**Estimate**		**Heterogeneity I2**	***P*-Value**
			**Prevalence**	**95% CI**	
Continents	Europe	6	0.19	0.09, 0.30	98.28	< 0.001
	Africa	2	0.05	0.04, 0.06	N/A	N/A
	Asia	3	0.32	0.13, 0.50	N/A	N/A
	North America	5	0.28	0.10, 0.45	99.64	< 0.001
	South America	2	0.10	0.08, 0.12	N/A	N/A
Study design	Cross-sectional	12	0.23	0.17, 0.29	99.26	< 0.001
	Cohort	5	0.26	0.03, 0.48	99.77	< 0.001
	Case-Control	1	0.36	0.23, 0.52	N/A	N/A
Type of drugs	Cocaine	9	0.27	0.08, 0.45	99.80	< 0.001
	Heroine	4	0.51	0.17, 0.88	99.75	< 0.001
	Opioids	8	0.35	0.08, 0.61	99.85	< 0.001
	PWID	11	0.17	0.10, 0.23	99.86	< 0.001
Stage Of TB	Active TB	16	0.22	0.16, 0.28	99.48	< 0.001
	Latent TB	5	0.34	−0.03, 0.72	N/A	N/A
	Active and Latent TB	2	0.21	0.18, 0.25	N/A	N/A
Imprisonment	Imprisoned TB/Drug	8	0.29	0.14, 0.45	99.63	< 0.001
	Not imprisoned TB/Drug	17	0.09	0.07, 0.12	97.58	< 0.001
TB-HIV	TB-HIV	17	0.14	0.11, 0.17	99.04	N/A

N/A, not mentioned.

- *WHO Regions*.

**Figure 4:**
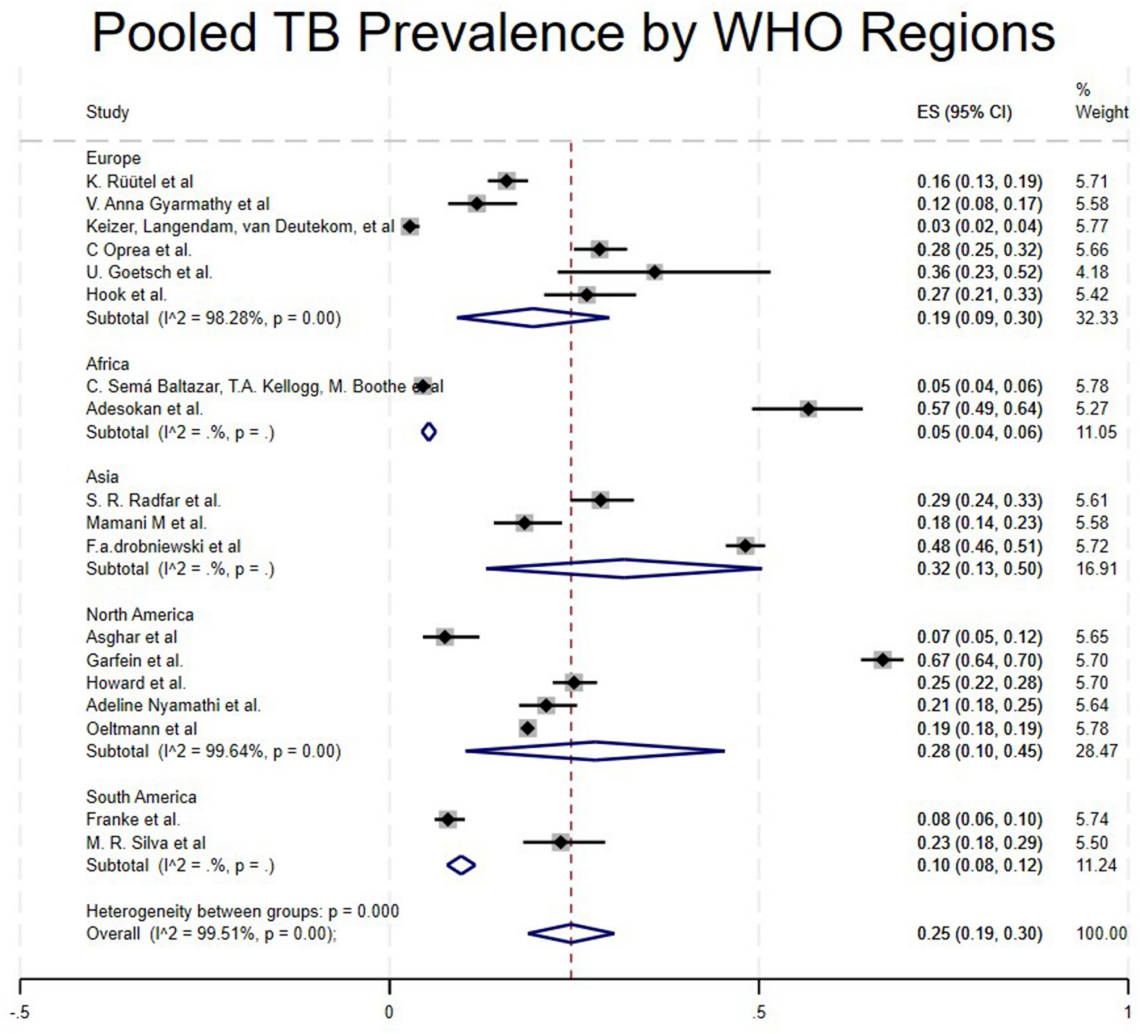
Pooled TB prevalence by WHO regions.

Studies from 5 regions met the inclusion criteria. The pooled prevalence varied significantly across regions. Studies conducted in Asia showed a high prevalence of TB among People who use drugs (PWUD) (32%, 95CI:13%−50%) (17, 22, 25), followed by North America with a pooled prevalence of 28% (95% CI: 10%−45%) (16, 21, 23, 24, 30), then Europe with a pooled prevalence of 19% (95% CI: 9%−30%) (5, 15, 18, 27–29), South America with a pooled prevalence of 10% (95% CI: 8%−12%) (31, 32) and Africa, which had the lowest pooled prevalence of 5% (95% CI: 4%−6%) (4, 26) as shown in Table 1 and Figure 4.

- *Types of Drugs*.

**Figure 5:**
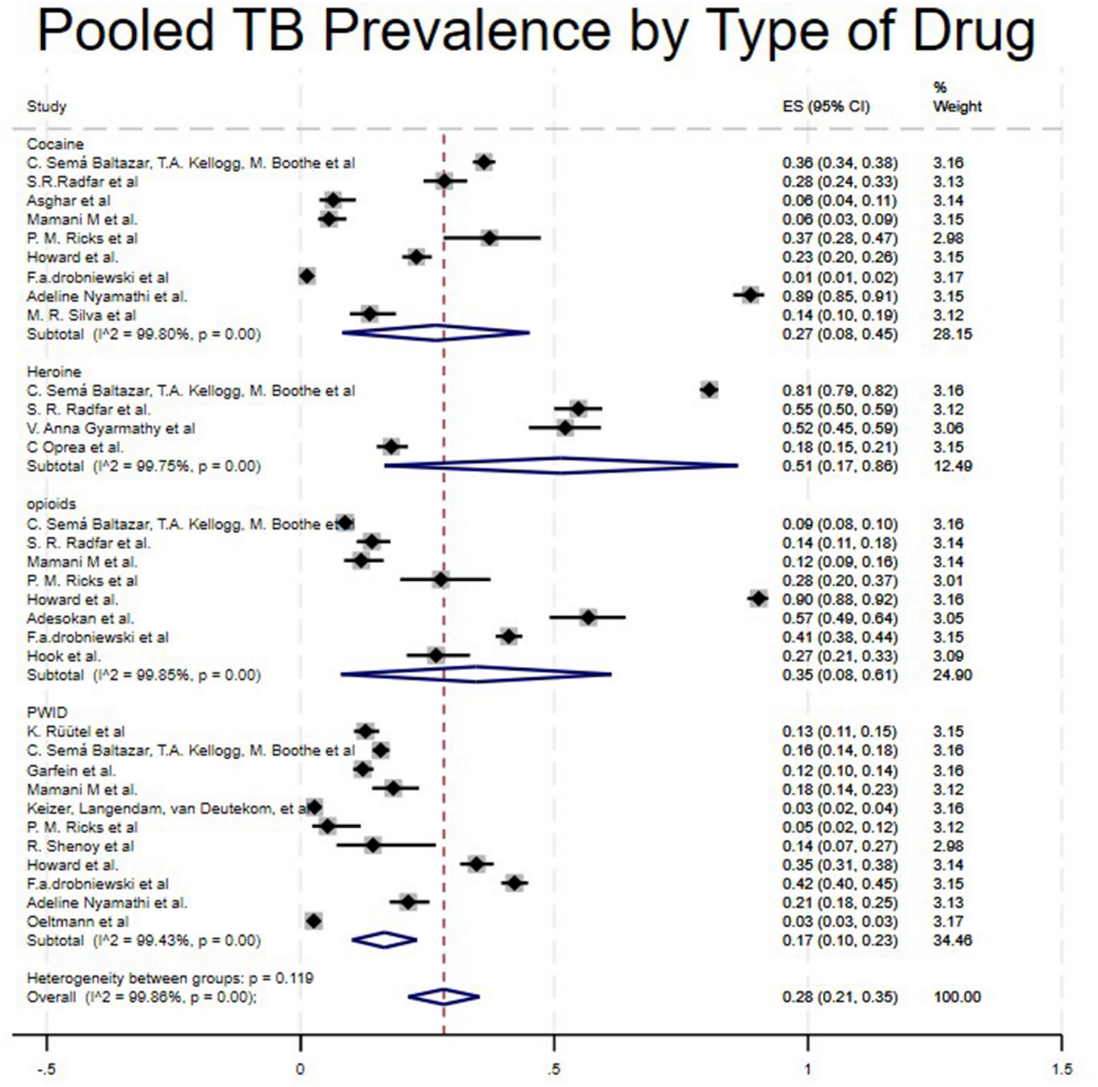
Pooled TB prevalence by type of drug.

Studies were categorized into four different drug types: Crack Cocaine, Heroin, Opioids, and PWID. Studies that included Heroin showed a high pooled prevalence of 51% (95% CI: 17%−86%) (4, 25, 28, 29), Opioids 35% (95%CI: 8%−61%) (4, 17, 19, 21, 22, 25–27), Crack Cocaine 27% (95%CI:8%−45%) (4, 17, 19, 21–23, 25, 30, 31) and PWID 17% (95CI:10%−23%) (4, 15–24). A study like (4), which included all kinds of drugs, showed different prevalence levels in cocaine, heroin, opioids, and PWID (36%, 81%, 9%, and 16%, respectively), as shown in Table 1 and Figure 5.

- *Stage of TB*.

**Figure 6:**
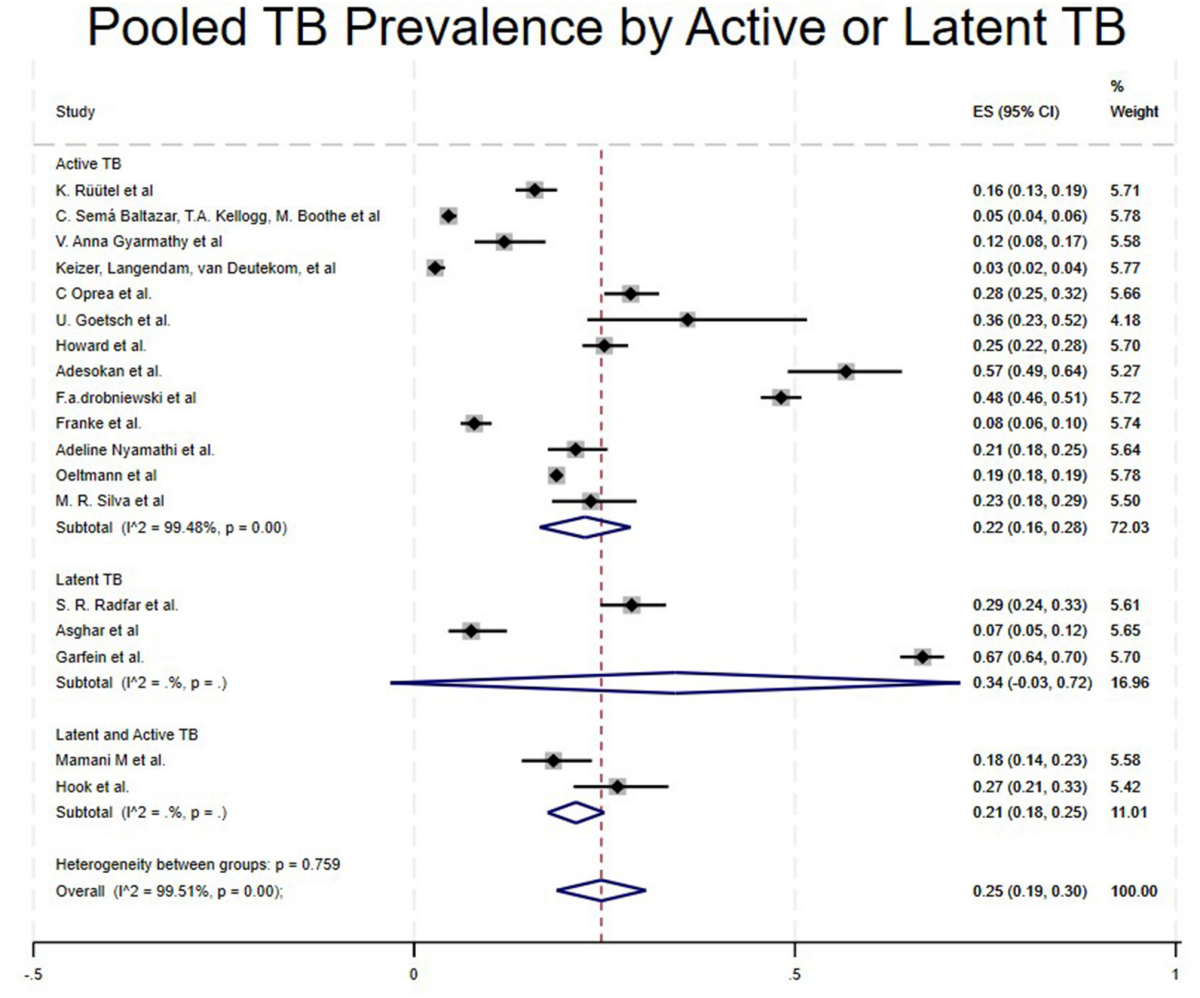
Pooled TB prevalence by active or latent TB.

Studies were categorized based on the stage of TB diagnosed, such as Latent TB, Active TB, and Both (Latent and Active TB). Studies that included Latent TB showed a high pooled prevalence of 34% (95% CI: −3% to 72%) (16, 25, 30), Active TB at 22% (95% CI: 16%−28%) (4, 5, 15, 18, 21–24, 26, 28, 29, 31, 32) and Both (Active and Latent TB) at 21% (95% CI: 18%−25%) (17, 27) as shown in Table 1 and Figure 6.

- *Imprisonment*.

**Figure 7:**
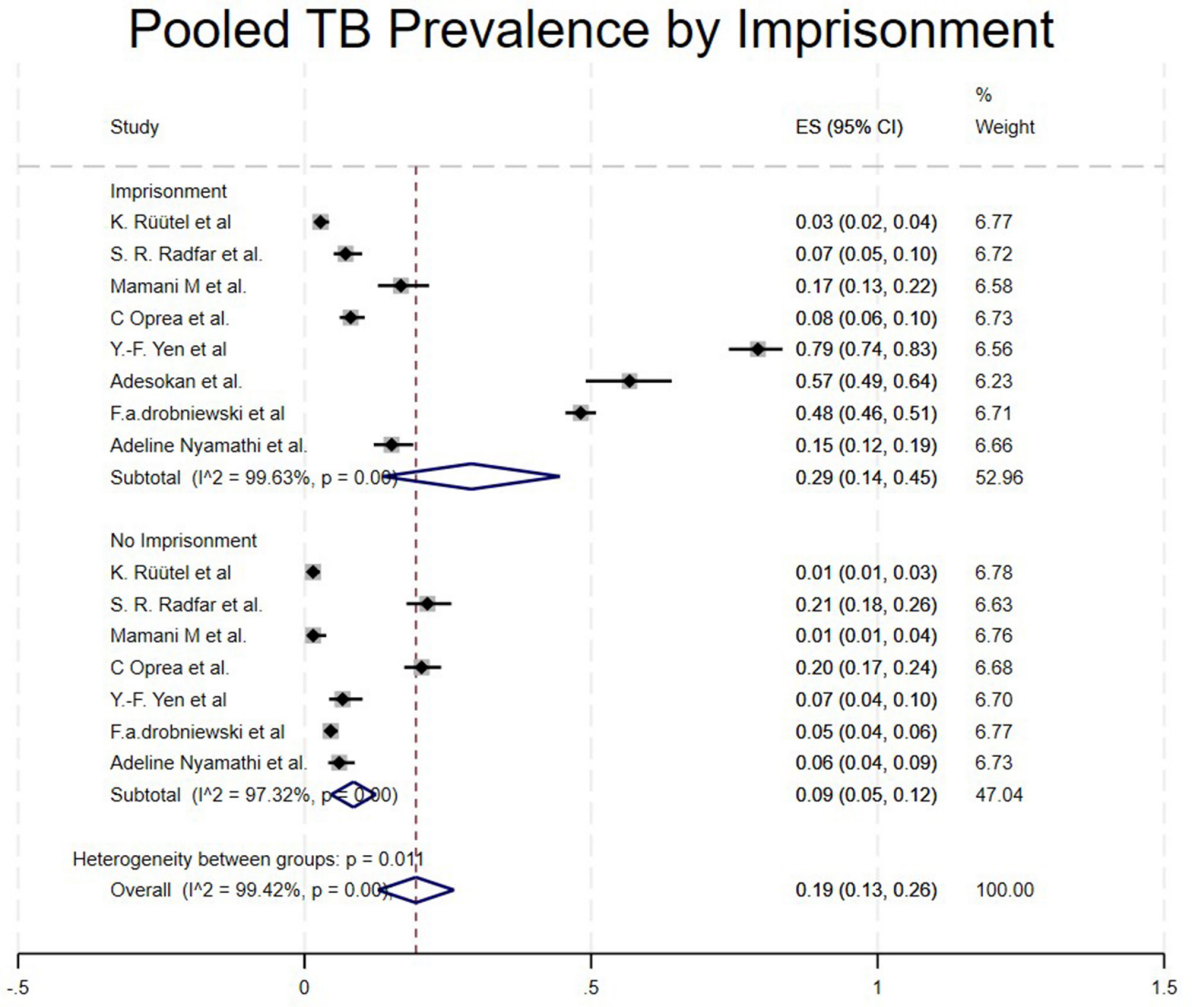
Pooled TB prevalence by imprisonment.

Studies were categorized based on whether the PWUD were imprisoned or not imprisoned, though they had TB. Studies that included imprisoned/TBPWUD showed a high prevalence of 29% (95% CI: 14%−45%) (15, 17, 22, 23, 25, 26, 29, 34), and not imprisoned/TBPWUD had a prevalence of 9% (95% CI: 7%−12%) (15, 17, 22, 23, 25, 29, 34), as shown in Table 1 and Figure 7.

- TB-HIV co-infection.

**Figure 8:**
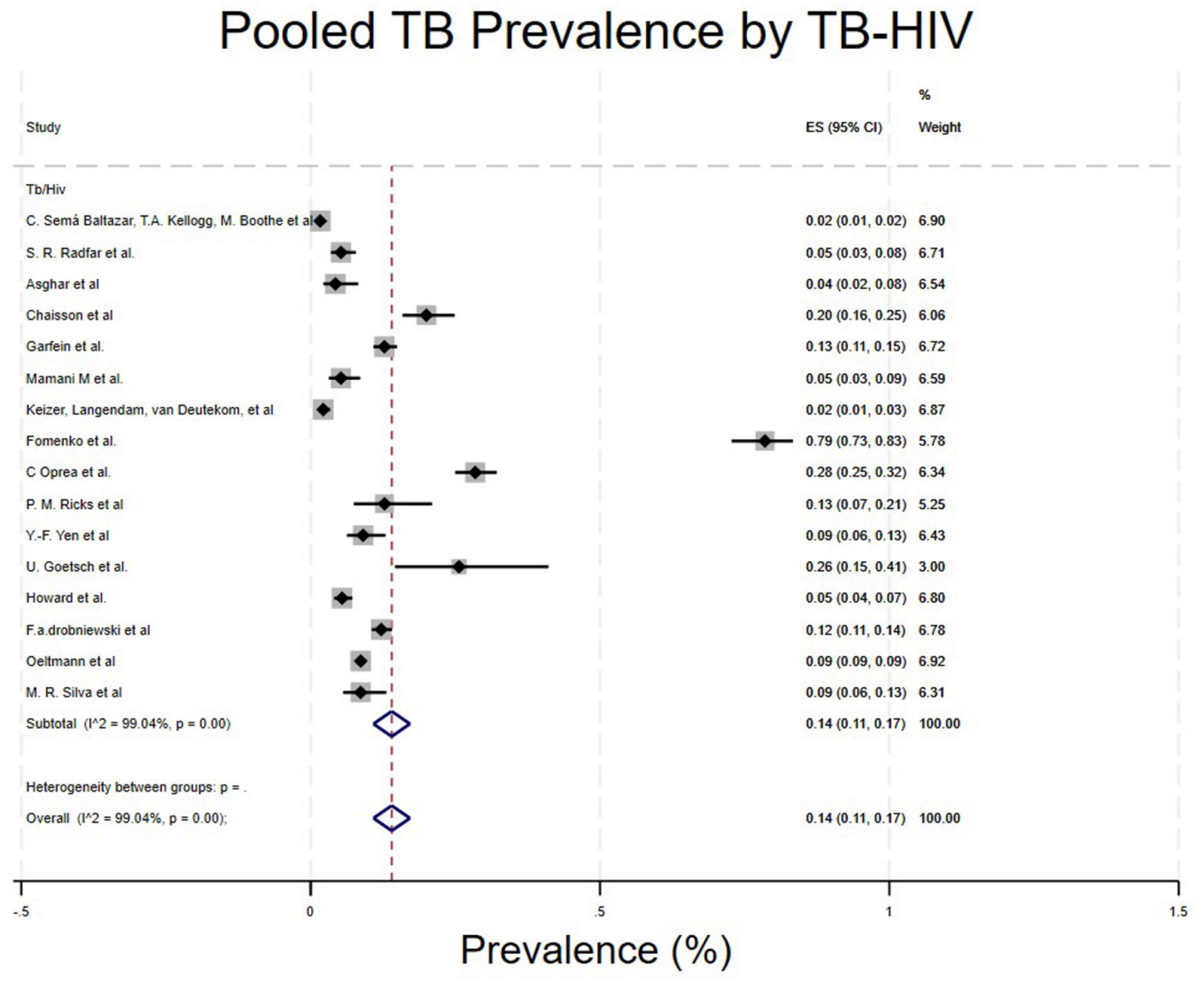
Pooled TB prevalence by TB-HIV co-infection.

Studies were categorized based on whether the PWUD had TB-HIV co-infection. Studies that included TB-HIV co-infection/PWUD showed a prevalence of 14% (96% CI: 11%−17%) (4, 5, 16–19, 21, 22, 24, 25, 29–31, 33–35) (Table 1; Figure 8).

### Quality evaluation

We used the modified Newcastle-Ottawa Scale (NOS) to evaluate the quality of the included literature, and the detailed evaluation results are shown in Table 2. Only four were evaluated as “very good” (9–10 points), 15 as “good” (7–8 points), and 4 as “Satisfactory” (5–6 points).

**Table 2:** Modified Newcastle—Ottawa quality assessment scale (adapted for Cross-sectional Studies).

**Included studies**	**Selection**			**Comparability**		**Outcome**			**Total score**
	**Representative of the Sample**	**Selection of the non-exposed cohort**	**Ascertainment of exposure**	**Demonstration that outcome of interest was not present at start of study**	**Comparability of cohorts on the basis of the design or analysis**	**Assessment of outcome**	**Was follow-up long enough for outcomes to occur**	**Adequacy of follow up of cohorts**	
Rüütel et al. (15)	Yes	Yes	Yes	Yes	Yes	Yes	N/A	N/A	Good
Baltazar et al. (4)	Yes	Yes	Yes	Yes	Yes	Yes	N/A	N/A	Good
Radfar et al. (25)	Yes	Yes	Yes	Yes	Yes	Yes	N/A	N/A	Good
Gyarmathy et al. (28)	Yes	Yes	Yes	Yes	Yes	N/A	N/A	N/A	Satisfactory
Oprea et al. (29)	Yes	Yes	Yes	Yes	Yes	Yes	Yes	N/A	Good
Asghar et al. (30)	Yes	Yes	Yes	Yes	Yes	Yes	Yes	N/A	Good
Ricks et al. (19)	Yes	N/A	Yes	Yes	Yes	Yes	Yes	yes	Very Good
Chaisson et al. (33)	Yes	Yes	Yes	Yes	Yes	Yes	Yes	N/A	Good
Shenoy et al. (20)	Yes	N/A	Yes	Yes	Yes	Yes	Yes	Yes	Good
Drobniewski et al. (22)	Yes	Yes	Yes	Yes	Yes	Yes	Yes	Yes	Very Good
Franke et al. (32)	Yes	N/A	Yes	Yes	Yes	Yes	Yes	Yes	Good
Goetsch et al. (5)	Yes	yes	Yes	Yes	Yes	Yes	Yes	N/A	Good
Nyamathi et al. (23)	Yes	N/A	Yes	N/A	Yes	Yes	N/A	yes	Satisfactory
Yen et al. (34)	Yes	N/A	Yes	Yes	Yes	Yes	Yes	N/A	Good
Adesokan et al. (26)	Yes	N/A	Yes	Yes	Yes	Yes	Yes	Yes	Good
Garfein et al. (16)	Yes	yes	Yes	Yes	Yes	Yes	Yes	Yes	Very Good
Howard et al. (21)	Yes	N/A	Yes	Yes	Yes	Yes	Yes	N/A	Good
Mamani et al. (17)	Yes	N/A	Yes	Yes	Yes	Yes	N/A	N/A	Satisfactory
Keizer et al. (18)	Yes	Yes	Yes	Yes	Yes	Yes	Yes	Yes	Very Good
Hook et al. (27)	Yes	Yes	Yes	Yes	Yes	Yes	Yes	N/A	Good
Fomenko et al. (35)	Yes	Yes	Yes	Yes	Yes	Yes	Yes	Yes	Good
Oeltmann et al. (24)	Yes	Yes	Yes	Yes	Yes	N/A	N/A	N/A	Satisfactory
Silva et al. (31)	Yes	N/A	Yes	Yes	Yes	Yes	Yes	N/A	Good

### Study populations and detection methods

An analysis was conducted on the patients included in 23 articles. The results showed that 16 articles exclusively enrolled patients with Active TB, 5 articles focused on Latent TB patients, and the remaining 2 articles included both populations. Although eight studies were conducted in settings where methadone was accessible (5, 17, 18, 21, 23, 33–35), the TB detection methods were categorized, except for the studies by Fomenko et al. and Hook et al., which did not specify the detection method; all other articles clearly described their methodologies. The most frequently used method was the TST (Tuberculin Skin Test), with detailed information presented in Table 3.

**Table 3:** TB stage classification and applied diagnostic testing.

**Author**	**Active or latent TB**	**TB test**
Rüütel et al. (15)	Active TB	IGRA
Baltazar et al. (4)	Active TB	Gene Xpert MTB/RIF
Radfar et al. (25)	Latent TB	TST
Gyarmathy et al. (28)	Active TB	TSH-Check 1
Asghar et al. (30)	Latent TB	Positive sputum smear
Chaisson et al. (33)	Latent TB	TST
Garfein et al. (16)	Latent TB	IGRA
Mamani et al. (17)	Latent and Active TB	TST
Keizer et al. (18)	Active TB	TST
Fomenko et al. (35)	Active TB	N/A
Oprea et al. (29)	Active TB	Positive sputum smear
Ricks et al. (19)	Active TB	TST and IGRA
Shenoy et al. (20)	Active TB	IGRA
Yen et al. (34)	Latent TB	TST
Goetsch et al. (5)	Active TB	TST
Howard et al. (21)	Active TB	TST
Adesokan et al. (26)	Active TB	Positive sputum smear
Drobniewski et al. (22)	Active TB	Positive sputum smear
Franke et al. (32)	Active TB	TST and IGRA
Hook et al. (27)	Active and Latent TB	N/A
Nyamathi et al. (23)	Active TB	TST
Oeltmann et al. (24)	Active TB	Positive sputum smear
Silva et al. (31)	Active TB	Positive sputum smear

### Publication bias

**Figure 9:**
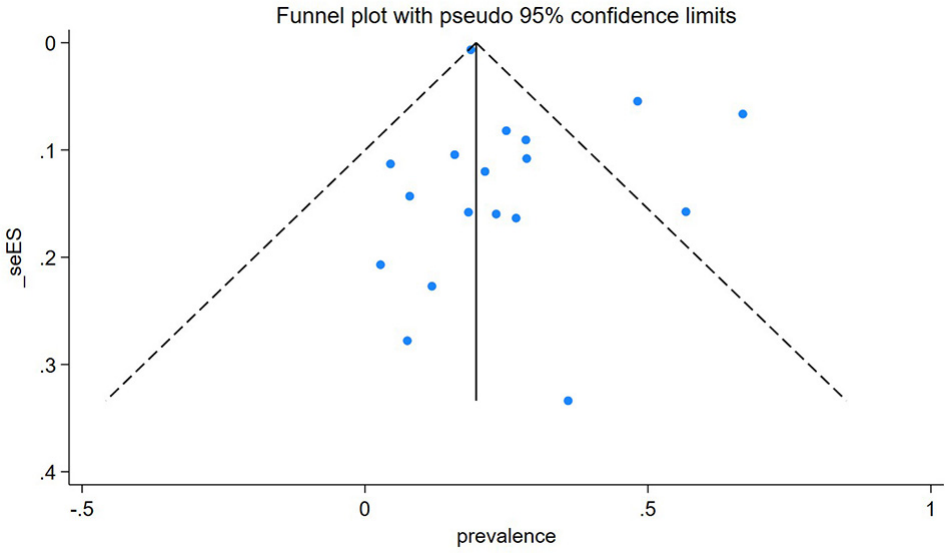
A funnel plot of prevalence against standard error.

Egger's publication bias plot is near the origin, and Egger's test p-value was (P = 0.146), indicating no publication bias for the prevalence of TB among People Who Use Drugs (PWUD). This is further supported by the symmetrical distribution on the funnel plot for TB prevalence among PWUD, as shown in Figure 9 alongside its standard error.

## Discussion

This study aimed to synthesize existing literature and estimate the prevalence of tuberculosis (TB) among People Who Use Drugs (PWUD). Our results show that the prevalence of TB among PWUD is 25% (95% CI: 19%−30%), meaning that one in four individuals who use drugs are likely to have active or latent TB. This prevalence is significantly higher than that of the general population (5–10%), highlighting the substantial burden of TB in this high-risk group (36).

Based on WHO regions subgroups, the highest prevalence of TB among People Who Use Drugs (PWUD) was seen in Asia at 32% (17, 22, 25), followed by North America (28%) (16, 21, 23, 24, 30). According to Rafiemanesh et al. (37), global data on TB epidemiology show a high prevalence among PWID. The positive TST rate ranges from 12% to 39% in North America, 17% to 52% in Europe, and just above 60% in Mexico. There is also a wide variation (from 0.5% to 66%) in reported active TB cases among PWID, depending on the study population and testing method. These differences are because the studies involved people who inject drugs. Injecting drugs triggers a rapid immune response and damages the immune system, making individuals more prone to infectious diseases like TB (17). This can increase susceptibility to Mycobacterium tuberculosis, leading to a higher risk of symptomatic infection.

In addition, drug use has been linked to poor treatment adherence, which worsens disease progression. Also, some participants included in the study were prison inmates. This could indicate they were exposed to poor hygiene and ventilation, increasing their susceptibility to infectious diseases like TB. Contrary to the overall statistics on TB prevalence, our study found a low rate in Africa (5%), while South America and Europe had rates of 10% and 19%, respectively (5, 15, 27–29, 31, 32). This difference may result from systemic issues like inadequate case detection caused by reliance on passive screening and less sensitive diagnostics (such as sputum smear alone), barriers to healthcare access worsened by stigma and criminalization of drug use, and sampling methods that don't accurately reflect the communities such as prison-based studies that overlook broader transmission patterns.

However, in the subgroup of TB diagnosis stages, we found that Latent TB had a higher pooled prevalence of 34% (16, 25, 30), followed by active TB at 22% (4, 5, 15, 18, 21–24, 26, 28, 29, 31, 32), and finally, both active and latent TB at 21% (17, 27). Our latent TB prevalence results seem slightly higher than those of the previous study conducted by Mamani et al., which reported a prevalence of 18.3% (17). This may be due to drug use, which is strongly associated with late diagnosis, along with epidemiological and environmental factors such as homelessness, tobacco and alcohol use, history of prison, and poor health status all of which may contribute to the high prevalence of latent TB among PWUD and increase their risk for TB infection.

Moreover, in our type of study subgroup, we found that there was a higher prevalence of TB among People Who Use Drugs (PWUD) in a case-control study (36%) (5), followed by cohort studies (26%) (16, 18, 29, 31, 32) and finally cross-Sectional Studies (23%) (4, 15, 17, 21–28, 30). This significant difference can be explained by the small number of studies included in the case-control subgroup. Having just one study might affect the validity of the estimate. Lastly, significant disparities based on drug types were observed. Heroin users showed a higher prevalence of 51%, followed by opioids at 31%, cocaine at 27%, and PWID at 17% (4, 5, 15–30, 32, 33, 35, 38). The variability in these results may be due to their administration methods, direct impact on the immune system, and relation to social determinant factors such as homelessness. Based on previous studies, heroin has been strongly associated with injection drug use, which has a high impact on immune suppression by damaging neutrophils and macrophages, key cells in controlling TB (35, 37).

Regarding the risk factors associated with TB among People Who Use Drugs (PWUD), our qualitative synthesis highlights that imprisonment (4, 16, 22, 26, 39) and homelessness (5, 23, 40, 41), as noted in Table 4 are significant contributors. Many sources indicate that the homeless population has a high incidence of tuberculosis linked to alcohol, tobacco, and illegal substance use, all of which are risk factors that promote the emergence of new cases and sustain the TB transmission cycle. Unemployment (21, 29, 40, 42, 43) is also associated with increased risk. A study conducted in Nepal found that unemployment increases the likelihood of not adhering to TB treatment (OR: 9.2). The study results show that unemployed individuals are less likely to complete therapy successfully. Conversely, a Malaysian study found no significant relationship between employment status and poor adherence to anti-TB therapy (44). However, the same study indicated that unemployed individuals had the highest rates of poor compliance compared to those with jobs. Defaulting therapies entails noncompliance, or failure to get treatment. 16 studies out of 23 highlighted how non-compliance to treatment will result to TB transmission increase (4, 5, 16, 18–20, 22–24, 27, 29, 31–35). Quantitative data on TB treatment completion among PWUDs reveal significant variability but underscore the potential for improvement with integrated care. Completion rates for drug-sensitive TB were reported as high as 79% in a trial setting with financial incentives (33) and 96% (23 of 24) in another cohort (18). However, a study in Ukraine reported a treatment success rate of just 52% for drug-sensitive TB and only 34% for drug-resistant TB (35). Crucially, this same study demonstrated that Opioid Substitution Therapy (OST) was associated with a statistically significant increase in treatment success for both drug-sensitive (61% vs. 42%) and drug-resistant TB (43% vs. 26%), highlighting OST as a critical determinant of positive outcomes. Previous research has also shown that tobacco smoking, as a predictor and risk factor, is strongly linked to ineffectiveness and a higher default rate in inadequate TB care settings. A study conducted in Hong Kong also found that smoking is a good indication of the probability of failing to complete TB treatment under DOTS (OR = 3.00, 95% CI 1.41–6.39, *p* = 0.004) (5).

**Table 4:** Factors that increase the risk of TB among PWUD.

**Factors that increase the risk of TB among substance abusers**	
**Social determinant health**	**Associated infection**
Imprisonment (2, 16–18, 34)	HIV and HCV (2, 17, 18, 22–24, 26, 31)
Homelessness (3, 28, 35, 36)	Default Treatment (4, 5, 16, 18–20, 22–24, 27, 29, 31–35)
Unemployment (19, 26, 35, 37, 38)	
Poverty (5, 6, 21)	
**Clinical aspect**	
Not BCG vaccinated (16, 27)	

The reviewed literature identifies a complex web of intersecting barriers that hinder treatment compliance, such as; Socioeconomic Factors like homelessness and substandard housing were consistently and significantly associated with treatment default and missed appointments (19, 23, 32), Drug Use and Mental Health; Drug use itself is a primary driver of poor adherence (23). Furthermore, this population exhibits a high prevalence of mental health distress, with over 70% reporting frequent sadness or depression and a history of suicide attempts, which worsens health outcomes and complicates care (27). Structural and Systemic Barriers: The high cost of treatment for complex cases (e.g., drug-resistant TB) does not guarantee success (29). Bureaucratic hurdles and a lack of coordinated care further impede progress. Immunological Impairment; Beyond behavioral challenges, Drug use causes direct immunologic impairments that increase the risk of progressing from latent to active TB disease, creating a more vulnerable patient population (24).

As seen in our study, imprisonment contributes highly to TB spreading among PWUD, with a prevalence of 29% (95% CI: 14%−45%) (17, 22, 26, 34). A study by Yen et al. highlighted that TB incidence in people who have been imprisoned is 4 times higher than that of those who haven't (34). Similarly, our study shows that imprisoned individuals have a higher prevalence of 29% compared to 9% of those not imprisoned. In Nigeria, imprisonment seems to increase TB spread due to overcrowded conditions, facilities with poor hygiene and ventilation (26). Additionally, co-infections such as HIV/AIDS are strongly linked to TB prevalence (4, 15, 16, 21, 22, 25, 28, 33). People Who Use Drugs (PWUD) are also susceptible to transmitting viral infections such as HIV, hepatitis B (HBV), hepatitis C (HCV), and other sexually transmitted diseases (STDs), primarily due to high-risk behaviors including needle sharing, exchanging sex for money or drugs, engaging with multiple sexual partners, low condom utilization, and inadequate vaccination coverage. (26, 27) and poverty (3, 7, 31) were related with TB among People who use drugs (PWUD).

Our analysis revealed significant unexplained heterogeneity across 23 studies, with subgroup analyses based on drug type, geography, and study design failing to explain the variability. Unmeasured factors such as healthcare access, HIV co-morbidity, or socioeconomic disparities in high-risk populations may account for these differences. These findings emphasize the importance of context-specific TB screening within drug use programs and standardized diagnostic reporting to enhance future research synthesis.

This study has several limitations. First, some subgroups included only a small number of studies, which can reduce the accuracy of the prevalence estimates and lead to potential under- or over-estimation of prevalence. Second, most studies lacked a control group, which might mask the true prevalence of tuberculosis among people who use drugs (PWUD) compared to the general population. Third, our study had notably high heterogeneity (*I*^2^ > 75%), which could not be resolved through subgroup analyses. Explored sources of heterogeneity included diagnostic methods (e.g., TST, culture, clinical criteria), definitions of TB (active vs. latent), and the way “People Who Use Drugs” (PWUD) are operationalized. This highlights that heterogeneity could be due to other unexplored factors. Fourth, only few studies were performed in Africa (with 2 studies) and South America (with 2 studies)—which limits the generalizability of the findings across these regions. Therefore, the observed low prevalence in these regions (5% and 10%, respectively) probably reflect data scarcity rather than true epidemiology. This obscures potential regional burdens, limiting region-based understanding of the risk factors. We recommend research in Africa and South America to explore more TB prevalence among PWUD. Despite these notable limitations, this study still highlights key findings that can inform policy decisions. The results underscore the importance of integrating TB screening and prevention into drug use programs and addressing systemic inequalities to reduce transmission. While TB treatment adherence among PWUDs is challenged by a syndemic of socioeconomic distress, drug use, and mental health issues, the evidence clearly shows that outcomes are significantly improved through integrated, patient-centered care models. Providing opioid substitution therapy is not just a harm reduction measure but a crucial part of successful TB control programs for this group. Future strategies must go beyond a solely medical approach to include structural interventions that address housing, mental health, and systemic barriers to care. Policymakers and public health professionals should systematically integrate services by colocating TB, harm reduction, and substance use programs, including TB case-finding in needle and syringe programs (NSPs), opioid agonist therapy (OAT) clinics, and drop-in centers; offering harm reduction measures and naloxone within TB clinics; and expanding infection control and LTBI treatment. They should also promote supportive environments through decriminalization and destigmatization, moving away from punitive drug laws, training healthcare and law enforcement personnel, and ensuring confidentiality and voluntary testing. Focusing on context-specific strategies and improving data collection in understudied regions will strengthen global TB control efforts. This is the first study to analytically assess the worldwide prevalence of tuberculosis among People Who Use Drugs (PWUD).

## Conclusion

This systematic review and meta-analysis provide the first global estimate of tuberculosis (TB) prevalence among People Who Use Drugs (PWUD), showing a pooled prevalence of 25% (95% CI: 19%−30%), with notable regional and methodological differences. Asia reported the highest prevalence (32%), likely linked to injection drug use and high-risk settings such as prisons. In comparison, Africa had the lowest (5%), possibly due to under-reporting or limited data. Study design and drug type also affected the estimates, with case-control studies and heroin users exhibiting the highest prevalence (36% and 51%, respectively). Key risk factors—including imprisonment, homelessness, unemployment, HIV co-infection, Default TB treatment, and poverty—highlight the complex socio-structural drivers of TB in this population. Despite some limitations, the findings of this study have significant policy implications. We recommend adding TB screening and prevention strategies into drug use programs, addressing systemic inequalities to reduce transmission, improving data reporting, and conducting further research in under-reported regions such as Africa and South Asia.

## Data Availability

The original contributions presented in the study are included in the article/Supplementary material, further inquiries can be directed to the corresponding author.
