# Supplementary material for: Prevalence of tuberculosis among People Who Use Drugs 2000–2024: a systematic review and meta-analysis
Source: Front Public Health. 2025 Oct 3;13:1635053. doi: 10.3389/fpubh.2025.1635053 (PMC12531209; doi:10.3389/fpubh.2025.1635053)
Supplement: Supplementary file 1 [file Supplementary_file_1.docx]

**Supplementary Table1: Search Strategy**

| **DATABASE NAME** | **SEARCH STRINGS** | **RESULTS** |
| --- | --- | --- |
| **PUBMED** | ("Tuberculosis"[Mesh] OR "Latent Tuberculosis"[Mesh] OR "Tuberculosis, Pulmonary"[Mesh] OR "Tuberculosis*"[tw] OR "Mycobacterium tuberculosis"[tw] OR "Pulmonary tuberculosis"[tw]) AND ("Substance Abuse Detection"[Mesh], "Substance Abuse, Intravenous"[Mesh], "Drug Users"[Mesh] OR "Drug abuse*"[tw] OR "Substance use"[tw] OR "Drug abusers"[tw]) | 457 |
| **WEB OF SCIENCE** | (“Tuberculosis*” OR “Mycobacterium tuberculosis” OR “Pulmonary tuberculosis”)) AND TS=("Substance Abuse Detection", OR "Substance Abuse Intravenous", "Drug Users" OR “Drug abuse*” OR “Substance use” OR “Drug abusers”) | 1345 |
| **SCIENCEDIRECT** | "Tuberculosis" "Pulmonary tuberculosis" "Drug abuse" "Substance use" "Drug abusers"  "Tuberculosis" "Pulmonary tuberculosis" "Drug abuse" "Substance use" "Drug abusers" | 44 |
