# Supplementary material for: Prevalence of tuberculosis among People Who Use Drugs 2000–2024: a systematic review and meta-analysis
Source: Front Public Health. 2025 Oct 3;13:1635053. doi: 10.3389/fpubh.2025.1635053 (PMC12531209; doi:10.3389/fpubh.2025.1635053)
Supplement: Supplementary file 2 [file Supplementary_file_2.docx]

Records identified from:

PUBMED (n =457)

Web of Science (n = 1,345)

Science direct (n = 283)

Records removed *before screening*:

Duplicate records removed (n = 387)

Records removed conducted before 2000yr (n = 443)

Records screened

(n =1,255)

Records excluded:

Titles and Abstracts Not Relevant to the Topic (n=1002)

Not in English (n =1)

Reports sought for retrieval

(n = 252)

Reports not retrieved

No Full Text (n = 21)

Reports assessed for eligibility

(n = 231)

Reports excluded:

Case Series and reports (n = 186)

No Clear Evidence (n =15)

No Clear Target Population Data (n =7)

Studies included in review

(n =23)

**Identification of studies via databases and registers**

**Identification**

**Screening**

**Included**

*Consider, if feasible to do so, reporting the number of records identified from each database or register searched (rather than the total number across all databases/registers).

**If automation tools were used, indicate how many records were excluded by a human and how many were excluded by automation tools.

Source: Page MJ, et al. BMJ 2021;372:n71. doi: 10.1136/bmj.n71.

This work is licensed under CC BY 4.0. To view a copy of this license, visit <https://creativecommons.org/licenses/by/4.0/>
